# Clinical guidelines for the management of craniofacial fibrous dysplasia

**DOI:** 10.1186/1750-1172-7-S1-S2

**Published:** 2012-05-24

**Authors:** JS Lee, EJ FitzGibbon, YR Chen, HJ Kim, LR Lustig, SO Akintoye, MT Collins, LB Kaban

**Affiliations:** 1 Department of Oral & Maxillofacial Surgery, University of California San Francisco, San Francisco, CA, USA; 2 Laboratory of Sensorimotor Research, National Eye Institute, National Institutes of Health, Bethesda, MD, USA; 3 Chang Gung University, Attending Plastic Surgeon, Chang Gung Craniofacial Center, Chang Gung Memorial Hospital, Taoyuan, Taiwan; 4 Head and Neck Surgery Branch, National Institute on Deafness and Other Communication Disorders, National Institutes of Health, Bethesda, MD and Department of Otolaryngology-Head and Neck Surgery, Georgetown University Medical Center, Washington, DC, USA; 5 Department of Otolaryngology-Head and Neck Surgery University of California San Francisco, San Francisco, CA, USA; 6 University of Pennsylvania School of Dental Medicine Department of Oral Medicine, Philadelphia PA, USA; 7 Skeletal Clinical Studies Unit, Craniofacial and Skeletal Diseases Branch, National Institute of Dental and Craniofacial Research, National Institutes of Health, Bethesda, MD, USA; 8 Walter C. Guralnick Professor and Chairman Department of Oral and Maxillofacial Surgery, Massachusetts General Hospital, Harvard School of Dental Medicine, Boston, MA, USA

## Abstract

Fibrous dysplasia (FD) is a non-malignant condition caused by post-zygotic, activating mutations of the *GNAS* gene that results in inhibition of the differentiation and proliferation of bone-forming stromal cells and leads to the replacement of normal bone and marrow by fibrous tissue and woven bone. The phenotype is variable and may be isolated to a single skeletal site or multiple sites and sometimes is associated with extraskeletal manifestations in the skin and/or endocrine organs (McCune-Albright syndrome). The clinical behavior and progression of FD may also vary, thereby making the management of this condition difficult with few established clinical guidelines. This paper provides a clinically-focused comprehensive description of craniofacial FD, its natural progression, the components of the diagnostic evaluation and the multi-disciplinary management, and considerations for future research.

## Definition

Fibrous dysplasia (FD) is a non-malignant condition in which normal bone and marrow are replaced by fibrous tissue and haphazardly distributed woven bone [1,2]. Patients may exhibit involvement of one bone (monostotic FD; MFD), multiple bones (polyostotic FD; PFD) or they may have McCune-Albright syndrome (MAS), which has been classically defined by the triad of PFD, café-au-lait skin macules and endocrinopathies, including among others, precocious puberty [3]. FD is caused by somatic activating mutations in the α subunit of the stimulatory G protein encoded by the gene *GNAS *[4,5]. A related disorder, cherubism, is manifest by expansile, multiloculated, radiolucent fibro-osseous lesions with multiple giant cells located bilaterally and symmetrically in the jaws. Cherubism is genetically distinct from FD and will be discussed elsewhere in the Proceedings of this meeting.

## Prevalence

**Figure 1:**
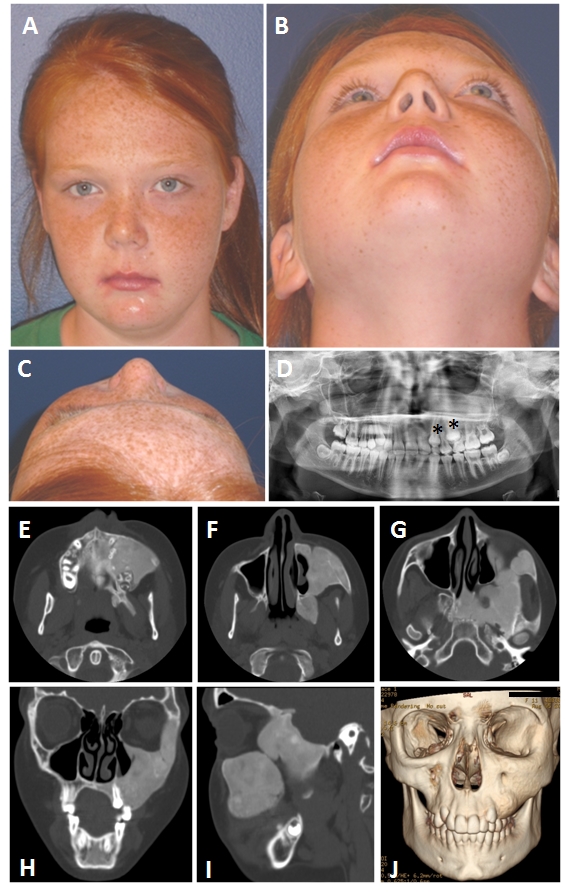
An 11-year old female with monostotic fibrous dysplasia of the left zygomatic-maxillary region. A-C) Clinical photographs demonstrating the appearance. The lesion was quiescent and asymptomatic. It had grown slowly over a period of years. D) Her dentist noted delayed eruption of her teeth (*) on that side as well as mild facial asymmetry and obtained the panorex that identified the lesion. E-I) CT images demonstrate the pathognomonic appearance of FD for her age, a homogenous, “ground-glass” lesion. J) The reconstructed CT image gives a sense of the three dimensional shape of the lesion that accounts for the clinical appearance.

**Figure 2:**
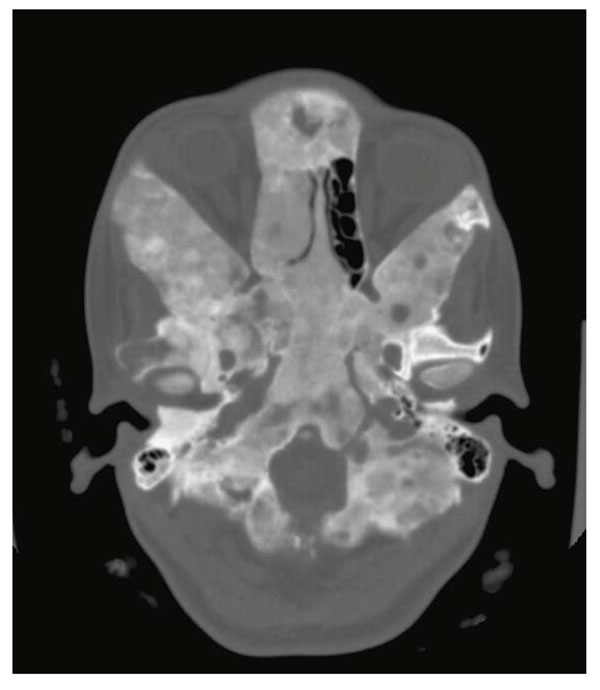
Extensive fibrous dysplasia involvement of the cranial base in a patient with MAS. In patients with PFD or MAS, the anterior cranial base is involved in 95% of the cases as seen in this CT image.

MFD is reported to be the most common manifestation of the disease, in some references it is estimated to occur four times more often than PFD [6]. However, in other series PFD is reported to be more common than MFD [7,8]. While the prevalence of MFD is probably greater than PFD, in none of the studies that define the relative prevalence of MFD versus PFD have the subjects undergone thorough skeletal and/or endocrine screening to determine the full extent of the skeletal and/or endocrine involvement. The most common locations are the craniofacial bones, proximal femur, and rib [2,8-11]. In MFD, the zygomatic-maxillary complex is reported to be the region most commonly involved (Figure 1A&B) [12]. In the less prevalent PFD and MAS, the craniofacial region is involved in 90% of the cases and the anterior cranial base is involved in over 95% of cases. (Figure 2) [13]. Depending on the type and location of FD, the signs and symptoms vary and include facial deformity and asymmetry, vision changes, hearing impairment, nasal congestion and/or obstruction, pain, paresthesia, and malocclusion. Many patients are asymptomatic and the diagnosis is made when a family member, friend or health care provider who has not seen the patient for a period of time notices asymmetry, or there is an incidental abnormality noted on dental or panoramic x-rays or on a head and neck computed tomogram (CT).

### Natural progression and clinical behavior

**Figure 3:**
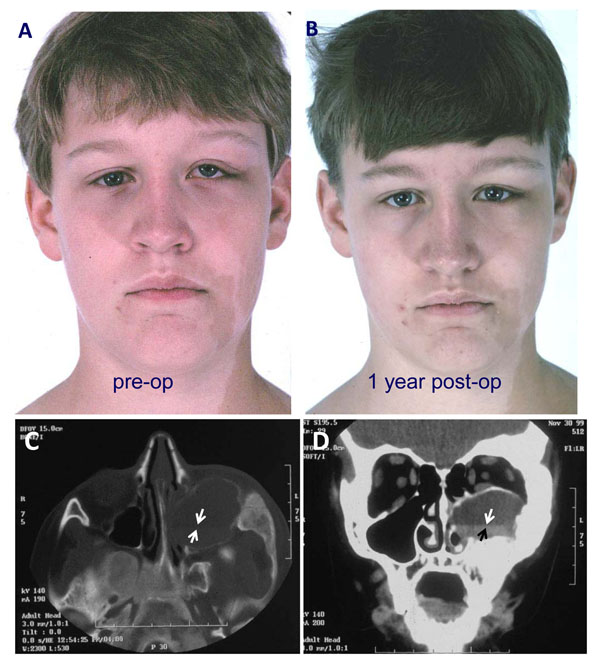
Fibrous dysplasia with a secondary aneurysmal bone cyst (ABC). A) The patient with a history of MAS complained of visual changes. Worsening asymmetry of the left eye and face was noted, and an on examination he was noted to have vertical dystopia of the orbit in the preoperative photograph. He was found to have a rapidly growing ABC within FD and underwent immediate resection and decompression of the ABC. B) The asymmetry and symptoms resolved after surgery. Note the classic café au lait spots of the left face and neck region as part of the triad of MAS. C&D) Preoperative CT images of the patient in A showing the FD lesion and associated ABC. Note the fluid/fluid level diagnostic of an ABC (arrows). The association of an ABC often results in aggressive behavior and rapid enlargement of the FD lesion with displacement of adjacent structures, in this case, the eye.

FD most commonly behaves as a slow and indolent growing mass lesion. The facial deformity and distortion of adjacent structures such as optic nerve, eye/globe, nasal airway, cranial nerve VII, middle ear ossicles, and teeth are gradual and insidious. Uncommonly, in young children and pre-pubertal adolescents, the lesions may demonstrate rapid growth, cortical bone expansion and displacement of adjacent structures such as the eye and the teeth. In some patients, rapid growth is associated with other pathological lesions such as aneurysmal bone cysts (ABC) or mucoceles (Figure 3) [13-15], or more rarely with malignant transformation. Malignant change to osteosarcoma or other forms of sarcoma has been reported to occur in less than 1% of cases of FD [16-22].

When rapid enlargement occurs, adjacent vital structures, such as the optic nerve, globe and auditory canal/structures and nasal airway may be invaded or compressed, resulting in functional deficits. For these reasons, some authors have advocated aggressive surgical resection to avoid potential blindness or hearing loss [23-26]. Rapid enlargement of FD in the nasal bones, maxilla or mandibular symphysis may result in airway obstruction by obliteration of the nasal cavity or by posterior displacement of the tongue. However, it has recently been demonstrated that such aggressive behavior with rapid expansion is the exception and that a conservative expectant approach is more prudent [13,14,27].

In MFD and PFD, progression of the lesions appears to taper off as the patients approach puberty (defined as skeletal maturity throughout this article) and beyond. Although continued active disease and symptoms into adulthood are uncommon, they have been reported [28-30]. In addition, in the NIH Screening and Natural History Study of Fibrous Dysplasia (SNHFD, protocol 98-D-0145) has documented persistent active disease and pain into adulthood in some patients. Based on >25 years of observation at the NIH, it appears that MFD, does not progress to PFD and neither progress to MAS [31].

In MAS, while growth of the lesions may also diminish after puberty, the overall degree of bony enlargement and deformity is often more severe and disfiguring than in patients with PFD. Data in the literature and observations by the NIH SNHFD indicate that the most severe deformities and symptoms occur in patients who have poorly controlled growth hormone excess [32-34]. It is recommended, therefore, that growth hormone excess in patients with PFD and MAS be aggressively managed.

In a retrospective study of 266 serial bone scans from 66 patients followed for up to 32 years in patients with extensive PFD or MAS, Hart et al. demonstrated that 90% of FD lesions, regardless of the site, were present prior to 15 years of age [31]. In the craniofacial region, 90% of all the lesions were detectable by bone scan by age 3.4, and no new lesions in the craniofacial region are very reported beyond the age of 10.

## Diagnosis and work-up

### Medical history and examination

A thorough history and physical examination are necessary to determine the extent of disease and to determine whether the FD is isolated or one of multiple lesions associated with PFD or MAS. Documentation of the onset and types of symptoms, presence of functional impairments and duration are imperative. Inquiries should include onset of menarche in females (to rule-out precocious puberty), other endocrine abnormalities or pathologies (such as hyperthyroidism, pituitary abnormalities, and renal phosphate wasting), growth abnormalities (review of growth charts), and history of fractures (to rule-out the presence of other FD lesions in the extremities) as well as the presence of skin lesions (café- au-lait lesions). These questions are particularly critical in young patients where underlying endocrine abnormalities may not have been detected and aggressive management is warranted. If there are any positive responses to the above inquiries, a referral to an endocrinologist is strongly recommended to rule out PFD or MAS. A skeletal survey or bone scan may be indicated if there is a suspicion of PFD or MAS, particularly in a patient that is not skeletally mature. Additional FD lesions beyond the craniofacial region require further evaluation by an orthopedic surgeon.

If the symptoms include rapid expansion, new onset of pain, visual change or loss, hearing change or loss, evidence of airway obstruction, new onset of paresthesia or numbness, a referral to a surgical specialist should be made immediately. Appropriate specialists that may be consulted include: neurosurgeons, craniofacial surgeons, oral & maxillofacial surgeons, otolaryngologists, neuro-ophthalmologists, audiologists and dentists, depending on the site of involvement or symptoms. In institutions where a craniofacial anomalies team is available, this may be an alternative referral that would assist the patient in further comprehensive evaluation.

## Imaging

CT imaging is recommended to define the anatomy of individual lesions and to establish the extent of disease. A standard craniofacial CT, without contrast and with slice thickness no greater than 3.75 mm (from top of the head to the thyroid region), is used to evaluate for the presence of FD in the skull base and facial bones. Historically, plain films of the craniofacial region were used but because of the overlapping of adjacent structures, involvement of the skull base was often underreported. For similar reasons, plain radiographs are not recommended for diagnostic purposes for cranial or facial lesions. Dental radiographs (i.e. panorex and dental films) or a cone-beam CT are appropriate to examine and help manage lesions around the dentition. Depending on the site of involvement, the appropriate referrals should be made for further analysis.

**Figure 4:**
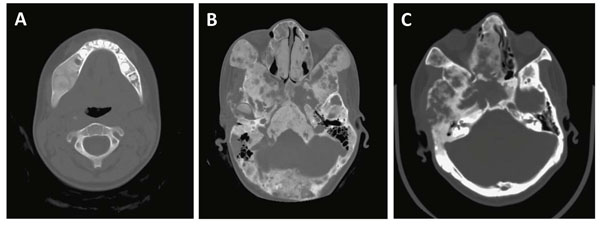
Variations in CT appearance of fibrous dysplasia based on age. A) FD in the young patient most often appears as homogenous, radio-dense lesions often described as having a ground glass appearance on CT. B) As these patients enter adolescence, the FD lesions progress to a mixed appearance which stabilizes in adulthood (C) but does not necessarily resume a homogenous appearance. This may explain the numerous radiographic descriptions of FD in the literature such as “ground-glass”, “pagetoid”, “lytic”, and “cystic”.

The most common radiographic characteristic of craniofacial FD is a “ground-glass” appearance with a thin cortex and without distinct borders [35]. In an ongoing study at NIH [36], it was demonstrated that the typical characteristics of FD on CT and the natural radiographic progression may vary from a “ground-glass” or homogenous appearance to a mixed radio-dense/radio-lucent lesion as the patient ages (Figure 4). In pre-pubertal patients with PFD or MAS, the lesions most often appear as homogenous, radio-dense lesions on CT. As these patients enter the second decade of life, the FD lesions progress to a mixed appearance, which stabilizes in adulthood but does not resume a homogenous appearance. While the change to a mixed radiographic appearance alone does not require further biopsy or investigation, we recommend careful monitoring and intermittent craniofacial CT during the pubertal phase of the young patient. This period of change in CT appearance coincides with case reports of increased activity of the FD lesions either through rapid growth, worsening facial asymmetry, malignant transformation, or association with other pathologic, radiolucent lesions such as an ABC and accelerated expansion [15]. Additionally, in our collective experience, there have been young patients who have the clinical and histologic diagnosis of a monostotic fibro-osseous lesion that are G_s_ mutation negative, yet demonstrate a rapidly enlarging and predominantly multi-loculated radiolucent appearance on CT and not the typical indolent growth. The exact pathophysiologic mechanism and its relationship to the variable genotype, i.e. is this a false negative gene test or another entity, has yet to be determined. If the patient is experiencing new onset of symptoms or rapid enlargement at *any* age, an updated CT is recommended as well as an immediate referral to the appropriate specialist for further investigation and management.

## Biopsy

A bone biopsy, by the appropriate surgical specialist, should be obtained to confirm the diagnosis of FD, if the site is amenable to biopsy. Unfortunately, the histology does not predict the biological behavior of these lesions [37,38]. Biopsy of FD does not specifically induce growth of the lesion. However FD lesions may be quite vascular and bleeding can be brisk. The surgeon should be prepared to deal with this. If the lesion is quiescent or asymptomatic, and/or in the cranial base, a biopsy may not be possible or necessary. History, clinical examination and the classic radiographic presentation are often adequate to establish the diagnosis of FD.

## Management by anatomic site and involvement

### Facial bones

**Figure 5:**
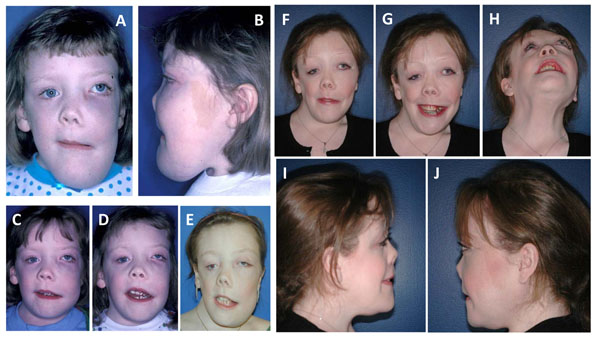
Serial images of a woman who presented at 9-year old with MAS and extensive fibrous dysplasia complicated by growth hormone excess. A&B) At presentation, she had a history of failure to thrive, airway obstruction, and was blind in the left eye at the time of presentation. Due to the airway obstruction in the nose and displacement of the tongue by the mandibular lesions, she underwent extensive contouring of the nasal bones, maxilla, and mandible with excellent results and patent airway. C-E) Over time, this patient’s lesions continued to grow but eventually stabilized by age 17 years. F-J) The patient 5 years after the second surgery. She has improved facial contours and symmetry though she continues to have pronounced orbital asymmetry. Her airway remains stable. She graduated magna cum laude from college.F) The 3D model of the patient demonstrates the enlargement of the maxilla, mandible, and blockage of the nasal cavity by the FD at age 17 years. G) The left mandible was significantly contoured to more normal proportions. H) Aggressive contouring of the left maxilla as well as the opening of the occluded nasal cavity. I) The nasal trumpet (green) was necessary to maintain a patent passageway while healing from surgery. J&K) Intraoperative view of the surgically removed fibrous dysplastic bone.

**Figure 6:**
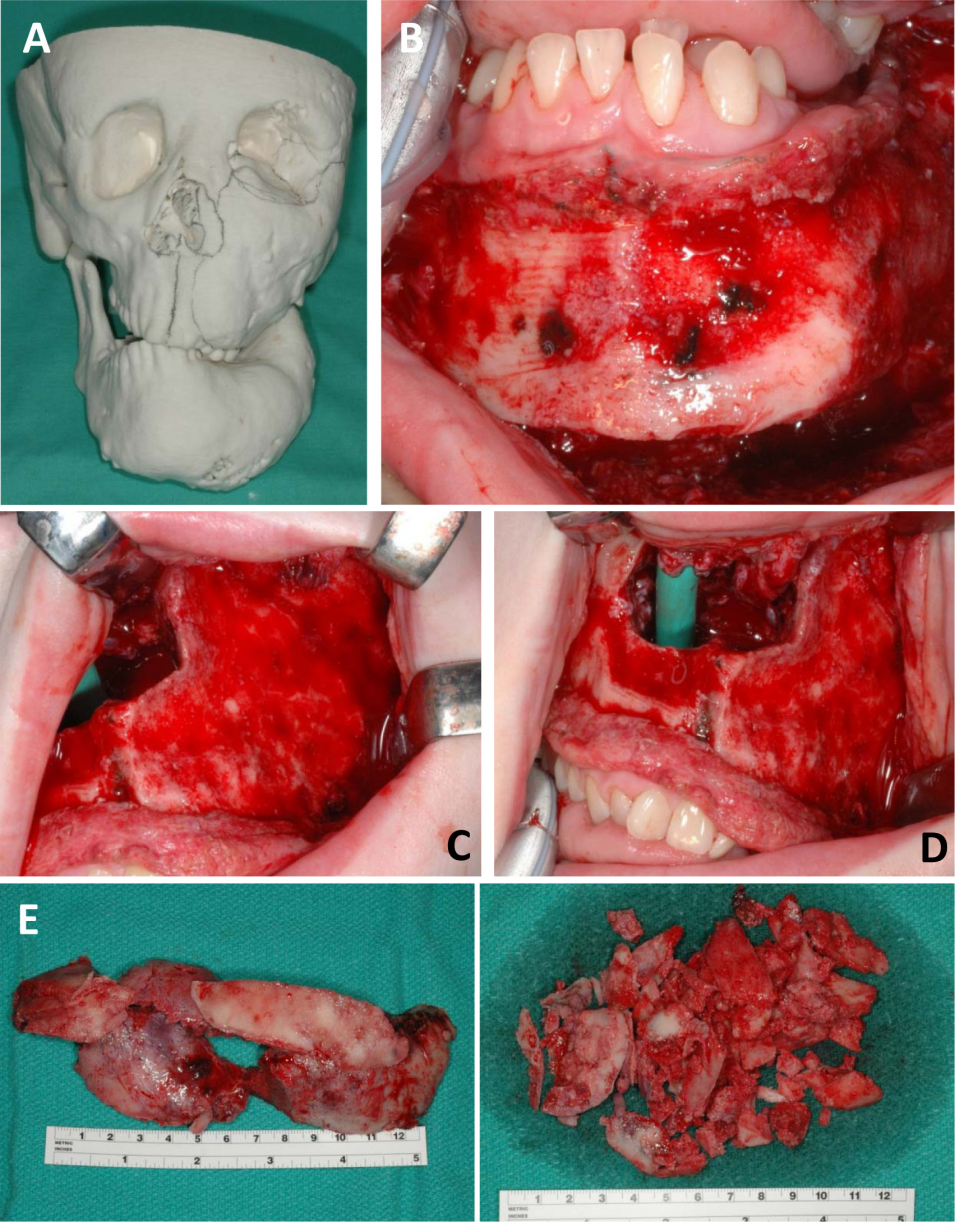
Serial images of the surgical approach to the woman from Figure 5 who presented at 9-year old with MAS and extensive fibrous dysplasia complicated by growth hormone excess. A) The 3D model of the patient demonstrates the enlargement of the maxilla, mandible, and blockage of the nasal cavity by the FD at age 17 years. B) The left mandible was significantly contoured to more normal proportions. C) Aggressive contouring of the left maxilla as well as the opening of the occluded nasal cavity. D) The nasal trumpet (green) was necessary to maintain a patent passageway while healing from surgery. E&F) Intraoperative view of the surgically removed fibrous dysplastic bone.

Asymmetry and swelling are the most common complaints when FD is found in the bones of the facial skeleton. Secondary deformities due to slow growing FD include vertical dystopia (difference in the vertical position of the eyes), proptosis, frontal bossing, facial and jaw asymmetries or canting. The degree of facial deformity varies, but those with MAS are the most severely affected, particularly when associated with untreated or inadequately treated growth hormone excess (Figure 5&6).

The diagnosis and management of facial lesions is at least in part based on the patient’s age and stage of skeletal maturity i.e. pediatric versus adult (skeletally mature). In the pediatric population, of all the patients who present for evaluation of facial swelling and asymmetry, more than half of all jaw tumors encountered are of mesenchymal cell lineage, and of these tumors nearly 50% are fibro-osseous lesions, a significant proportion of which are FD [37,39]. Thus, FD must be high on the differential diagnosis for children with facial swelling and asymmetry. The management of FD in young and older patients is dictated by the clinical and biological behavior of the lesion, as the histology does not provide reliable prognostic or predictive information. There are currently no biomarkers to predict the behavior of these fibro-osseous lesions [37]. This is particularly concerning in pediatric patients because of the potential for active growth, malignant transformation and association with other tumors.

The FD lesions of the face may be described as quiescent (stable with no growth), non-aggressive (slow growing), or aggressive (rapid growth +/- pain, paresthesia, pathologic fracture, malignant transformation, association with a secondary lesion). In the case of a quiescent FD lesion in which the patient does not complain of facial deformity, observation and monitoring for changes is an acceptable treatment modality. Annual evaluations may be adequate. The patient’s concerns and symptoms, clinical assessment including sensory nerve testing in the region of involvement, photographs, and facial CT should be obtained at each visit. An annual CT may be necessary for the first 2 years; however, the interval may be lengthened based on the clinical findings. Surgical contouring by a maxillofacial or craniofacial surgeon is indicated if the patient is bothered by facial disfigurement. While complete resection may be possible in monostotic lesions, it is unlikely to be possible in PFD or MAS), and the surgeon must weigh the reconstruction options that will provide the patient with the best outcome as well as preserve the function of adjacent nerves and structures. These patients may also require orthognathic surgery to correct a concurrent malocclusion or facial/dental canting [40]. There is no documented contraindication for orthognathic surgery so long as the lesions are quiescent. Bone healing appears to be normal with conventional rigid fixation [40]. Regular follow-up with the surgeon is necessary to determine that there is no recurrence and further deformity.

In patients with non-aggressive but active FD, it is ideal to wait until the lesion becomes quiescent and the patient has reached skeletal maturity before performing an operation. However, in cases where the patient’s psychosocial development may be impaired due to the facial deformity, surgical contouring and/or resection may be warranted. The patient and family must be aware of the potential for regrowth if the lesion cannot be resected completely, which is often the case. In cases of PFD or MAS where the disease is extensive, the lesions are often not resectable. Repeat surgical contouring and extensive debulking may be necessary to achieve acceptable facial proportions [41]. In the future, improvement in CT imaging and software will allow for accurate surgical simulation and intraoperative navigational tools may guide the surgeon throughout the contouring. Advanced CT software is useful for superimposition of pre- and post-operative images. These can then be compared to follow-up CT scans to determine stability of the result or the presence of regrowth. Despite these new imaging technologies, there is no therapy or technology that can predict and/or prevent regrowth.

Patients with aggressive and rapidly expanding FD, occasionally complain of new onset pain or paresthesia/anesthesia [15]. Based on the site of involvement, the patient may also report visual disturbances, epiphora, impaired hearing, nasal congestion or obstruction, sinus congestion and pain and malocclusion. We recommend immediate evaluation by a maxillofacial surgeon, ENT, or craniofacial surgeon and CT imaging. The etiology of this change in behavior may not be readily identified but documented causes include: associated expansile lesions such as ABC or mucocele, malignant transformation, and osteomyelitis. A biopsy of the area of growth is necessary prior to surgical management. Treatment may range from contour resection to en bloc resection depending on the diagnosis.

In cases of an associated lesion, the management is based on that associated lesion e.g. an ABC with FD would warrant curettage of the ABC and contouring of the underlying FD.

Malignant transformation of FD has been reported in less than 1% of cases of FD [16-22]. Typically the malignancy is a sarcomatous lesion, most often osteosarcoma but fibrosarcoma, chondrosarcoma, and malignant fibrohistiocytoma have also been reported [16,20,28,42-45]. The diagnosis may be difficult, particularly in cases of low-grade osteosarcoma [46,47]. In such cases, immunohistochemical analysis with MDM2 and CDK4 may assist in distinguishing FD from a malignancy as a malignancies will often express MDM2 or CDK4 while FD will not [48,49]. The treatment is based on the management of the malignancy and resection with adequate margins is necessary.

Osteomyelitis must be treated with prolonged antibiotic therapy and consultation with an infectious disease specialist. The limited literature and our collective experience indicate that osteomyelitis in the setting of FD is difficult to diagnose and to successfully treat [50-54]. We have managed patients that developed osteomyelitis of the jaws after attempts at exposure and orthodontic movement of impacted teeth. It may resolve with prolonged antibiotic treatment and pain management, however en bloc resection of the FD lesion may be required for refractory pain and persistent infection.

## Sinuses

**Figure 7:**
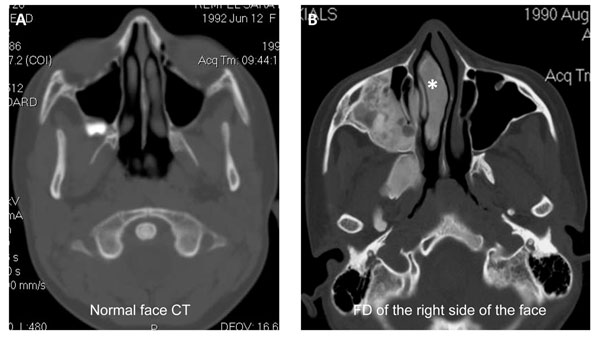
Fibrous dysplasia involving the right maxillary sinus and turbinate. A) Normal facial CT without any FD for comparison. B) FD in the right maxilla and extension into the maxillary sinus. There is also FD involvement of the right turbinate (*) that may explain the patient’s nasal congestion.

The sinuses may be affected by FD, with the most frequent site being the sphenoid sinus, followed by the ethmoid and maxillary sinuses (Figure 7) [55]. This is not surprising, as the anterior cranial base is often affected in patients with craniofacial PFD [13]. The entire sinus can be completely obliterated by FD, yet surprisingly the incidence of sinusitis is not greater than the general population in these patients. This may be explained by the loss of air space and Schneiderian membrane in an obliterated sinus and the elimination of a source of infection. Patients typically complain of nasal congestion (>34% of those with symptoms and sinus involvement), headaches or facial pain, recurrent sinusitis, and hyposmia. This appears to be associated with FD in the inferior turbinate and the subsequent hypertrophy. There appears to be a correlation between nasal congestion and hyposmia and the severity of disease, but a history of sinusitis and facial pain/headaches does not correlate with the amount of craniofacial disease [55]. The findings by DeKlotz and Kim also note that growth hormone excess is associated with more significant involvement of the sinonasal region [55].

The management of sinus and nasal congestion includes nasal saline spray, nasal steroid spray, antihistamines for those with seasonal allergies, and antibiotics for suspected bacterial sinus infections. Consultation with an otolaryngologist may be necessary for persistent congestion and chronic sinus infections. Though there is very little literature on the effectiveness of sinus surgery in patients with FD sinus disease and sinus obliteration, if surgery is indicated, we recommend waiting until the adjacent FD is quiescent and the patient is at least in the late teens and skeletally mature to minimize the possibility of regrowth and necessity for re-treatment. Endoscopic sinus surgery with and without image-guided systems has become a popular approach [56-58], although it may be necessary to combine endoscopy with a traditional external approach [59,60]. The extent of resection should be based on the location of the pathological bone and its proximity to important sinus structures, as radical or complete resection may not be necessary or possible. The effectiveness of endoscopic surgery for FD is undetermined as sinus surgery is not commonly done in patients with FD.

The association of other expansile lesions such as a mucocele or ABC with sinus FD may result in rapid growth of the combined lesion [61,62]. This is particularly concerning in areas adjacent to the skull base and brain such as the sphenoid, ethmoid, and frontal sinuses where access may be limited. The symptoms depend on the adjacent involved structures such as the eye, optic nerve, crista galli, and brain. A referral to a multidisciplinary skull base surgery center is necessary for further evaluation and treatment.

## Teeth

The dental variations in FD and the management of dental problems in patients with FD are poorly characterized. Due to the lack of information, the dental community is wary of treating patients with FD or MAS out of concern for potential post-procedure complications and exacerbation of the FD lesions around the teeth [63].

**Figure 8:**
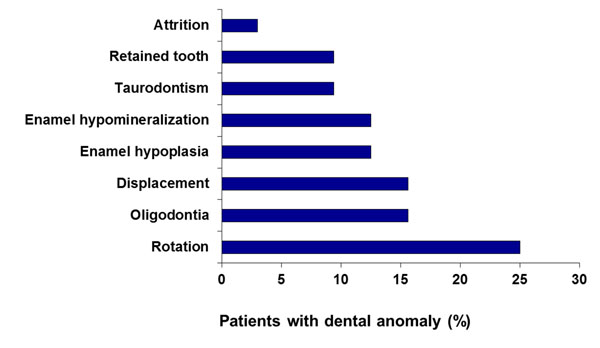
Dental anomalies seen in patients with fibrous dysplasia of the jaw bones. In a study by Akintoye et al [64], 41% of the patients with FD had dental anomalies in general and 28% of the patients had the dental anomaly within FD bone. Adapted from reference [64]

Akintoye et al [64] examined 32 patients with craniofacial FD that were enrolled in the SNHFD Study. Twenty-three patients had PFD/MAS and 9 had monostotic disease; this population reflected the NIH study population with more extensive disease. In this study, 41% of the patients had dental anomalies in general, and 28% of the patients had the dental anomaly within FD bone. The most common anomalies included: tooth rotation, oligodontia, displacement, enamel hypoplasia, enamel hypomineralization, taurodontism, retained deciduous teeth, and attrition (Figure 8). There was no correlation between any endocrine dysfunction or renal phosphate wasting and enamel hypoplasia or hypomineralization, attrition, or any of the other tooth anomalies. However, taurodontism, a condition noted on dental radiographs characterized by enlargement of the pulp chamber in multi-rooted teeth, has been described in patients with syndromes including growth hormone excess [65,66] but never in FD/MAS. Taurodontism was noted only in the FD patients that had 1 or more endocrinopathies. While taurodontism does not require special dental care, it may be an indicator of an underlying endocrinopathy associated with MAS.

The caries index scores were higher among FD patients (Table 1). This may be attributed to the increased enamel hypoplasia and hypomineralization or the limited dental care these patients receive. There were no histological abnormalities in the extracted wisdom teeth that may explain the increased caries index scores. We recommend more frequent dental visits, every 3-4 months. Additionally, no patients reported any complications or exacerbation of their FD lesions after dental restorations, tooth extractions, orthodontic therapy, odontoma removal, maxillary cyst removal, or biopsy of the jaws. Among the 10 patients that received orthodontic therapy, the duration of treatment appeared somewhat longer than conventional cases (2-4 years in duration), the results were less than satisfactory, and there was relapse. We recommend careful monitoring of the post-orthodontic results in patients with FD. Despite the extensive disease in and around the dentition in some of the patients, the arch form was predominantly maintained without significant displacement of the teeth as compared to other benign growths.

**Table 1:** 

Caries Index		
	Fibrous dysplasia DFT scores	Normal DMFT values*

4-17 years	2.9	1.7

≥18 years	9.6	6.6

*W.H.O country oral health profile-USA DMFT

While this may describe the natural progression of most FD, there is clearly a subset of patients that have the clinical and histologic diagnosis of FD that have rapid growth of the facial lesions, radiolucent changes on CT, and the displacement of teeth from the natural arch form. While some of these lesions have tested G_s_α mutation negative, many patients in this subset have not been genetically characterized to determine if the absence of the G_s_α mutation in the presence of a fibro-osseous lesion increases the risk of aggressive behavior and aberrant growth. Further studies are necessary to discern the implications of the mutation or lack of the mutation.

For patients with missing teeth, dental endosseous implants may be considered [67]. Bone healing and integration of the implants occurs, though it may be slower and the quality of bone is consistent with grade 3 or 4 bone as the cortex is often thin or nonexistent. In a reported case of a 32-year old female with MAS, successful integration and loading of dental implants in the maxilla and mandible occurred. The maxillomandibular lesions had been quiescent for 3 years. The dental implants were at least 15 mm in length and were functional after 5 years. The literature is limited, and it is unclear whether there is an increased risk of implant failure. There is also the concern that osteomyelitis may occur in the setting of a failed implant. If implant treatment is considered, we recommend that the implant be placed once growth of the FD lesion has subsided. Additionally, we would recommend following the principles of implant placement and place the dental implants after a young patient has completed growth to avoid submerged implants and revision of the prosthesis [68].

## Skull base disease

### Orbit/optic nerve/sphenoid bone

**Figure 9:**
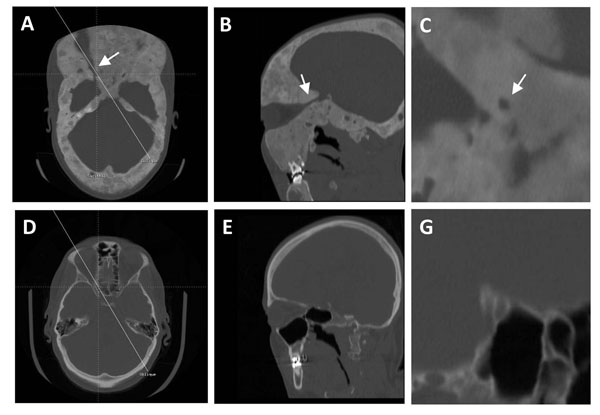
Fibrous dysplasia encasing the optic nerve compared to a normal optic canal. A-C) A patient with extensive fibrous dysplasia (FD). The arrow indicates the optic canal. D-G) CT of a normal and uninvolved optic canal. Several CT slices through the optic canal are shown: A&D) axial, B&E) oblique, and C&G) coronal. A case-control study by Lee et al [13] demonstrated that statistically significant narrowing of the optic canal by FD did not result in vision loss. Thus, observation with regular ophthalmologic examinations in patients with asymptomatic encasement was a reasonable treatment option and optic nerve decompression was not warranted. Adapted from reference [13]

Common findings associated with PFD around the eye include proptosis, dystopia, and hypertelorism due to the involvement of the frontal, sphenoid, and ethmoid regions [30,69]. Less common findings include: optic neuropathy, strabismus, lid closure problems, nasolacrimal duct obstruction and tearing, trigeminal neuralgia and muscle palsy with skull base involvement [70,71] (FitzGibbon, unpublished data). There has been significant controversy regarding the management of FD of the sphenoid bones that encase the optic nerve, particularly in patients whose vision is normal (Figure 9). Clinicians have assumed that such encasement seen on CT will cause blindness because of the proximity and compression of the optic nerve by FD, and because of reported cases of acute loss of vision. In one study it was reported that vision loss was the most common neurologic complication in this disease [72]. With such concerns in mind, prophylactic decompression of the optic nerve (“unroofing”) has been recommended by many surgeons [23-26]. Unfortunately, decompression may result in no improvement of vision (reported in 5-33% of cases), or worse postoperative blindness. In addition the abnormal bone tends to grow back in most cases. The first case-control study was conducted by Lee et al [13] to evaluate a cohort of patients with extensive cranial base FD, and determined that observation with regular ophthalmologic examinations in patients with asymptomatic encasement was a reasonable treatment option and optic nerve decompression was not warranted. Though there was statistically significant narrowing of the optic canal in patients with FD, this did not result in increased vision loss and there was no correlation between the findings on the CT and the neuro-ophthalmologic exam. These findings were confirmed by Cutler et al. in a study that included an analysis of the same group of subjects after longer follow-up together with an initial analysis of additional subjects [33]. A recent meta-analysis that included, in addition to the most recent analysis of the NIH SNHFD cohort, an analysis of all the published cases of optic nerve decompression surgery, came to the same conclusions [73]. Based on these results, we recommend that FD in the skull base around vital structures, including the optic nerve, should be managed according to the clinical examination and regular diagnostic imaging and observation is appropriate in asymptomatic patients [13,27,33,73,74].

**Figure 10:**
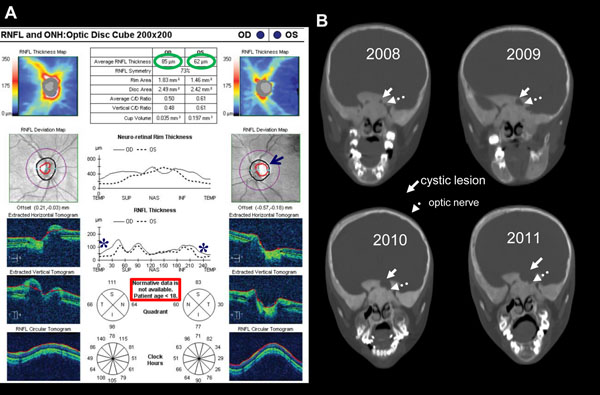
A representative Cirrus^TM^ optical coherence tomography (OCT) optic disc cube study (A) and serial CT scans (B) of a 9-year-old girl with subtle left optic neuropathy and a very slowly expanding cystic lesion abutting the left optic canal. A) The numbers in the two green circles in the RNFL (retinal nerve fiber layer) represent the single number comparison between the two eyes. Generally, the nerve fiber layer is considered thin when it is less than about 70 microns. Note that in the RNFL Deviation Map panels the optic cup (the area within the red circle) on the left (OS) (black arrow) is a bit larger than on the right (OD), also suggestive of axon loss. In the RNFL Thickness graph, note the differences between the left (dashed line) and the right (solid line) in the temporal (TEMP) region (asterisk), indicating that in this region retinal nerve fibers are thinner on the left. For children under 18 normative data for the Extracted Vertical Tomogram and the RNFL Tomogram are not available. B) Serial coronal plane CT images at approximately the same region are shown. The expansile cystic lesion is indicated with the solid white arrow, and the optic nerve by the dashed arrow. The findings indicate the presence of a slowly expanding lesion, the cystic, fluid-filled nature of which was confirmed on MRI. On clinical examination, there were subtle findings of left optic neuropathy in that she performed slightly worse on the Ishihara color test and the Pelli Robson test of contrast sensitivity in her left eye. There was no evidence of an afferent pupil defect. Photos also demonstrated subtle temporal pallor of her left optic disc. There were no objective changes in visual acuity. She has been followed clinically with neuro-ophthalmologic examination approximately every three months to assess for any significant progression, which would be an indication for surgical intervention. The findings on the OCT study confirm the clinical impression of a left optic neuropathy and are particularly useful when visual fields are not obtainable or particularly reliable (usually due to age-related inability to perform the test), as well as an objective measure for longitudinal follow-up. The nerve fiber layer findings on OCT can also be used to predict what visual outcome one might expect after a successful decompression surgery. If one were to find a field defect on examination, but the corresponding optic nerve retinal nerve fiber layer was preserved on OCT testing, it would be reasonable to expect full recovery of vision after surgery. However, if there were nerve fiber layer loss, recovery of vision would be unlikely as the findings most likely represent axons that have died back.

Once it is determined that there is FD surrounding the optic nerve(s) and orbit, a comprehensive neuro-ophthalmologic examination should be done to establish the baseline. This should be followed by comprehensive annual exams. The exam should concentrate on assessing for optic neuropathy and include visual acuity, visual-field exam, contrast sensitivity, color vision, and dilated fundus exam. Additional examination should include pupillary examination for afferent pupil, extraocular movements, proptosis measurement with exophthalmometry, lid closure, hypertelorism, and tear duct and puncta exam. The diagnosis of optic neuropathy should be reserved for those with a visual field defect or if 2 of the 3 exams (contrast sensitivity, color vision, and fundus/disc exam) are abnormal. A new diagnostic modality, optical coherence tomography (OCT), uses high resolution cross-sections of the optic nerve to determine the thickness of the retinal nerve fiber layer (RNFL) [75-79]. A thin RNFL correlates with visual field changes and evidence of optic neuropathy. This modality may be useful for examining patients that cannot undergo a visual field exam (such as children) or may predict visual recovery after surgery. In the case where the RNFL may be thin prior to surgery, it is unlikely that surgery will improve vision while a patient with a normal RNFL may have some improvement after surgical treatment (either decompression or proptosis correction). A representative case of the utility of the combination of OCT, clinical examination, and imaging is shown in Figure 10.

The etiology of the visual changes and vision loss in patients with craniofacial FD remains unclear. However, patients with abnormal findings are more likely to have an associated endocrinopathy, most commonly growth hormone excess, which typically results in gradual loss of vision, if vision loss is observed. In the cases of other lesions such as an aneurysmal bone cyst or mucocele, vision loss can be much more rapid. A study by Cutler et al [33] demonstrated that 12% of patients with relatively severe craniofacial PFD had evidence of optic neuropathy, that patients with GH excess had a higher relative risk for complete encasement of the optic nerve (4.1 fold), and had a higher relative risk for optic neuropathy (3.8 fold) compared to patients without GH excess. Preliminary findings by Glover et al demonstrated that patients with an early diagnosis and treatment of GH excess had no optic neuropathy (0 of 14 patients that were diagnosed and treated by age 18) while 4 of 7 patients diagnosed and treated for growth hormone excess after age 18 had optic neuropathy [80]. We strongly recommend that patients with craniofacial PFD are evaluated for growth hormone excess or MAS and that if endocrinopathies are present they be aggressively managed.

Patients with acute visual change or vision loss should undergo a CT of the cranial base and immediate referral to a neurosurgeon or craniofacial surgeon and neuro-ophthalmologist. Several case reports have noted the association of a new, expansile lesion near the optic nerve, typically an aneurysmal bone cyst, and high dose glucocorticoids with immediate decompression and resection is indicated [15]. Unfortunately, the success of surgical treatment is unknown due to the limited cases of acute vision loss.

## Auditory canal/temporal bone/cranial nerves

**Figure 11:**
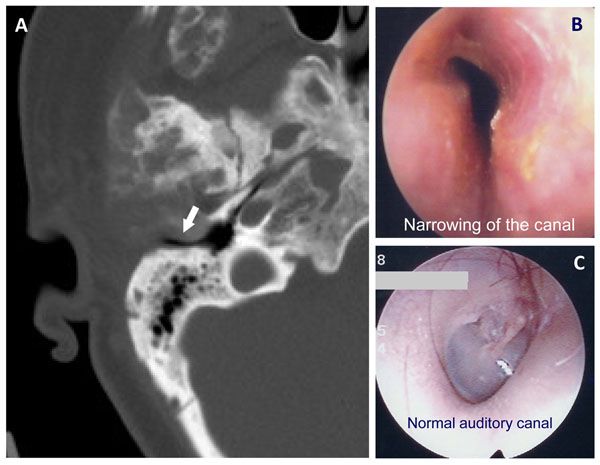
Narrowing of the external auditory canal due to fibrous dysplasia (FD). A) A CT image of a coronal slice through the temporal bone shows a narrowed external auditory canal (arrow) B) Narrowing of the canal is shown and can be compared to a normal canal in (C). The arrow on the CT image (A) demonstrates narrowing of the canal. This has resulted in hearing loss. The clinical images on the right compare a canal narrowed by FD to a normal external auditory canal.

**Figure 12:**
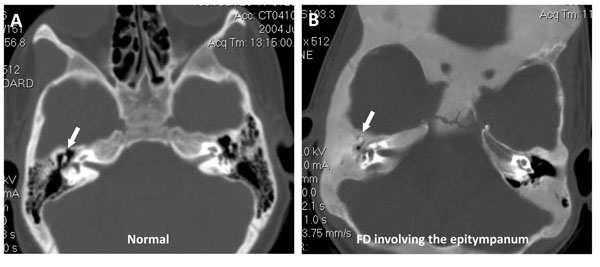
Fibrous dysplasia of the epitympanum compressing the ossicles. The arrows point to the ossicles in a normal epitympanum (A) and in a FD-involved epitympanum (B). The crowding in the right image has resulted in hearing loss.

The temporal bone is frequently involved (>70%) in patients with craniofacial PFD or MAS [81], while temporal bone involvement is uncommon in monostotic disease [82,83]. In a recent analysis by DeKlotz et al., despite the high incidence of disease of the temporal bone in PFD, nearly 85% of patients had normal or near-normal hearing; 10% had conductive hearing loss due to PFD, approximately 4% had sensorineural or mixed hearing loss (both conductive and sensorineural), and the remainder had hearing loss due to other, non-PFD related causes. In most cases, the degree of hearing loss was mild (77%) and did not correlate to the amount of disease involvement of the temporal bone. The common causes of hearing loss appeared to be narrowing of the external auditory canal due to the surrounding FD (Figure 11) and fixation of the ossicles within the epitympanum from adjacent involved bone (Figure 12). The narrowing of the external auditory canal may result in significant cerumen buildup. Therefore, it is recommended that regular otolaryngology exams are performed to maintain patency in patients in whom the external auditory canal is particularly narrowed. A rare but potentially concerning complication is the development of a cholesteatoma, an obstruction of the canal with cerumen and desquamated skin [83,84]. This complication typically requires surgical intervention to relieve the obstruction and chronic infection [82,85]. In the case of PFD or MAS, there is concern that contouring and excision of the surrounding FD may exacerbate regrowth of the lesion. However, only case reports have been documented noting this possibility.

We recommend a comprehensive audiology examination and ear evaluation once the temporal bone is found to be involved with FD. Annual hearing/audiology exams are recommended during the active bone growth. For external auditory canal stenosis, regular exams under microscopy are usually required by the otolaryngologist. Surgery for the external auditory canal is recommended for complications such as cholesteatoma or near total ear canal stenosis; however it may be beneficial to wait until growth has slowed and the patient has progressed beyond puberty.

Temporal bone involvement may also result in facial nerve weakness or paralysis as the CN VII exits the cranium through the petrous temporal bone. This finding is quite rare and is likely caused by the compression of the cranial nerve within the Fallopian canal and/or the internal auditory canal [71,83,86,87]. Unfortunately, the location of the compression may be extremely difficult to access. In case of sudden facial weakness, a high resolution cranial base or temporal bone CT is indicated. If an expanding mass within the FD is noted, a referral to a skull base surgeon is warranted for consideration of surgical decompression.

## Nonsurgical and adjuvant management of craniofacial FD

While pain is common among FD patients, [88], there are very few studies with a detailed assessment of the symptoms and there is a need for more data relating pain to the location and activity of disease and the effectiveness of various treatment modalities. Kelly et al [11] examined 78 patients (35 children and 43 adults) and found 67% complained of pain. It was not uncommon for the pain to be undertreated; some patients required NSAIDs with and without narcotic treatment, and others were treated with bisphosphonates. Interestingly, the pain scores did not correlate with the disease burden, and adults were more likely to have pain and have more severe pain than children, suggesting there is an age-related increase in the prevalence of pain in FD. They also noted that, despite the high prevalence of craniofacial FD, less than 50% had pain in the craniofacial region, in contrast to at least 50% of patients with lower extremity disease, another high prevalence site, complained of pain. In the same study, approximately 20% of the patients were managed with bisphosphonates and nearly 75% reported pain relief or improvement with this class of drugs.

The use of bisphosphonates such as alendronate, pamidronate, or zoledronic acid for craniofacial FD has been considered for pain reduction and to reduce the rate of growth of the lesion. In general, the clinical studies have demonstrated mixed results on the efficacy of bisphosphonates and FD-related pain with small sample sizes and with most studies examining all skeletal regions, not just the craniofacial sites. Plotkin et al [89] examined 18 children and adolescents with PFD or MAS and initiated IV pamidronate therapy. They found that pain seemed to decrease (not quantified) and serum alkaline phosphatase and urinary N-telopeptides decreased. There were no serious side effects from the bisphosphonate use however they noted no radiographic or histomorphometric change or improvement of the FD lesions. Matarazzo et al [90] reported on 13 patients with MAS who were treated with pamidronate for 2-6 years, and found a decrease in long bone pain, lowered fracture rate and bone turnover markers, and an increase in bone density on DEXA scan. Chan et al [91] followed 3 children with MAS for 8-10.5 years who were age 2.5-5 years at the start of treatment with pamidronate for MAS. They too noted a decrease in long bone pain and fracture rate however the long bone lesions continued to expand and grow while the facial lesions did not expand; there was no encroachment on the optic nerve throughout the follow-up. Chao et al [92] noted that oral alendronate over a 6-month course reduced intractable headaches and relieved the 3 patients from analgesic dependence. They reported no tumor progression, however the 3 patients were adults and may not have shown progression without the bisphosphonate treatment. Further studies are necessary to determine the efficacy osteoclast inhibitor therapies such as bisphosphonates or denosumab in slowing the growth of craniofacial FD and reducing intractable craniofacial FD pain. The variation in response between children and adults with FD and the safety of prolonged bisphosphonate use in children also require more investigation. New therapies are emerging that include RANK ligand inhibition (i.e. denosumab) however at this time their role in the treatment of FD-related pain or reduction in growth remains to be determined [93].

## Conclusion

We have provided the current understanding of the biologic and clinical characteristics of FD and recommendations for the clinical management in the craniofacial region. Most importantly, each patient may present with variable symptoms and clinical findings, thus the care of these patients must be customized to their needs and sites of involvement.

## Recommendations

1. Aggressively screen for and manage endocrinopathies (particularly growth hormone excess).

2. Active disease (rapid growth, new onset of pain or paresthesia, visual or hearing changes) warrants an immediate surgical referral and evaluation.

3. A bone biopsy should be obtained if there is any doubt about the diagnosis. If the lesion is in a site that cannot be biopsied due to unacceptable risks, history, clinical examination and radiographic diagnosis may be adequate for diagnosis.

4. Postpone surgical treatment of lesions until after skeletal maturity when the lesion is quiescent.

5. Surgical resection or contouring may be warranted prior to skeletal maturity if there are symptoms or rapid change in the lesion, however, patients must be aware of the risk of regrowth.

6. Potential use of adjuvant therapy such as bisphosphonates may be considered for refractory pain at the FD site.

7. Management of patients with FD, particularly PFD and MAS, requires a comprehensive evaluation and multi-disciplinary involvement for optimal care.

## Research questions

1. What are the mechanisms for changes in FD that occur as patients age?

2. What is the mechanism and effect of growth hormone excess on the growth rate and activity of FD?

3. What are potential targeted therapies and mechanisms that can be used to treat FD?

4. What biomarkers might be useful to predict biological behavior and growth of FD lesions?

5. What potential biomarkers or predictors of transformation and associated pathologies can be developed?

6. What combined therapies will prevent recurrence and regrowth (e.g. an operation with adjuvant bisphosphonates, interferon)?

7. What pharmacologic or molecular therapies may reverse the effects of the abnormal gene products in FD?

8. Does the detectability of a G_s_ mutation in a fibro-osseous lesion predict clinical behavior?

9. Is mutation testing a necessary component of FD evaluation?

## Competing interests

The authors declare that they have no competing interests
